# Integrating laboratory networks, surveillance systems and public health institutes in Africa

**DOI:** 10.4102/ajlm.v5i3.431

**Published:** 2016-10-31

**Authors:** Philip C. Onyebujoh, Ajay K. Thirumala, Jean-Bosco Ndihokubwayo

**Affiliations:** 1 WHO AFRO TB Laboratories Focal Point, World Health Organization, Harare, Zimbabwe; 2 Independent Consultant, Horamavu, Bangalore, India; 3 Lab Systems, WHO Africa Regional Office, Brazzaville, Congo

## Abstract

The Ebola outbreak in West Africa underlined the urgent need for integration of public health systems, including the establishment of national laboratory networks, surveillance systems, and health research institutions at all levels of service delivery. The integration schema presented here would assist in driving the immediate steps needed for integration of public health systems, particularly laboratory networks, in support of the implementation of International Health Regulations and the Global Health Security Agenda in the African region. Increased funding, political willingness from countries, and coordination through enhanced technical assistance from international partners, are critical in achieving this objective.

## Introduction

The recent Ebola virus disease crisis in three West African countries – Guinea, Liberia and Sierra Leone – highlighted several concerns with respect to the functioning of public health systems in the resource-constrained settings of Africa.^1,2,3^ Key concerns included: (1) lack of capacity for timely clinical screening, referral, diagnosis, notification, treatment and containment of people with suspected infection; (2) lack of effective surveillance and response systems and trained workforce; (3) rapid breakdown of existing public health services due to the Ebola virus disease outbreak; (4) inadequate investment in building integrated, cross-cutting systems with the capacity to respond to public health emergencies; (5) challenges in implementing the World Health Organization’s (WHO) International Health Regulations (IHR); and (6) insufficient research and development capacity at the country and regional levels for new drugs, diagnostics and treatment strategies. These concerns and challenges are not limited to the three countries that experienced the recent crisis. In general, challenges faced by public health systems in Africa, especially at the primary care level, include: (1) lack of laboratory facilities integrated across diseases and services; (2) poor funding and uptake of new rapid diagnostic technologies; (3) overemphasis on resourcing vertical disease-control programmes; (4) patient and health system delays; and (5) lack of fully-integrated electronic disease surveillance systems.

## Laboratory structures, role of public health institutions and need for integration

Laboratories play a critical role in the prompt diagnosis of diseases and treatment monitoring. The specific administrative structures of public health laboratories within Ministries of Health differ among countries in Africa. In the majority of countries in the African region, laboratories fall under a directorate, department or unit for national laboratory services, which are housed within the Ministry of Health. Difficulties in coordination of laboratory services with disease control programmes have been observed in such conventional administrative structures. District and below district-level hospital laboratories act as the default ‘integrated’ laboratories, due to inadequate staff levels, skill sets and infrastructure. Intermediate-level laboratories are practically district-level laboratories and act as regional laboratories, but without the necessary functional dichotomy. Primary health centre laboratories are poorly equipped for rapid diagnosis or rapid response; and national-level laboratories are not fully networked with district-level laboratories for the functional management of laboratory networks.^4^ Donors and partners in several countries promote ‘parallel’, disease-specific laboratories, especially for the major diseases of poverty (tuberculosis, HIV and malaria), as governments fail to address deficiencies. In a few countries, government-run centres-of-excellence were developed as public health research institutes with support from international partners.^5^ In some countries, environmental health protection and food safety services, as well as national drug testing laboratories, are also administered under public health services.

The lack of adequate resources, both financial and technical, has contributed to undue prioritisation of key diseases, which has in turn resulted in vertical disease control programmes and laboratories (e.g., tuberculosis, malaria and HIV). The absence of the integration of policy formulation, strategy and budgeting have been compounded by competing disease funding priorities and has created disjointed laboratory services in several developing countries.^6^ This has resulted in disparate and loosely-organised skill deployment, resource wastage and redundancies within the system. Laboratory workers with the cross-cutting skills to effect integrated services have been systematically under-utilised, as they are increasingly engaged in services for single-disease programmes.^7^

Some of the key issues plaguing public health laboratories^8,9^ include: (1) under-resourced infrastructure, including equipment; (2) poor laboratory linkages to clinical services, resulting in low test demand and suboptimal utilisation of modern diagnostics for clinical decisions; (3) poor laboratory quality control and assurance systems; (4) a paucity of leadership to provide adequate policy intervention, technical guidance and supportive supervision; and (5) inadequate or absent national laboratory networks. These issues impact staff motivation and the overall credibility of laboratory services.

In spite of the functional challenges in the delivery of services, there is an urgent need to integrate already-existing public health technical capacity within countries in the African region. Effective linkages between public health institutes and laboratories, in addition to optimisation of services, would facilitate early detection of emerging antibiotic resistance, cancers and other non-communicable diseases, as well as periodic epidemiological disease surveys, which would inform policy changes.

The Network of African National Public Health Institutes^10^ works to facilitate the collaboration and strengthening of public health research institutes affiliated to the Network of African National Public Health Institutes in Africa.^11^ Present membership of the network comprises 16 institutions, including the Kenya Medical Research Institute, the Uganda Viral Research Institute, and the South African National Institute for Communicable Diseases. In addition, in some countries in Africa, private-sector clinical pathology laboratories, either corporate chains, or individual stand-alone laboratories, offer services to people who can afford quality care for a premium (such as medical aid/insurance, or out-of-pocket expenses). Systematically engaging African national public health research institutions and centres of excellence, as well as private-sector laboratories, with national laboratory systems and integrated surveillances systems would enhance utilisation of available laboratory and research capacities, and management of resources.

## Integration of public health institutions and laboratories through the implementation of International Health Regulations and the Integrated Disease Surveillance and Response framework

In 2005, the IHR mandated countries to detect, assess and respond to all events that may constitute public health emergencies of international concern, and report the events to the WHO.^12^ The Integrated Disease Surveillance and Response (IDSR) programme, a comprehensive regional framework for strengthening national public health surveillance and response systems in Africa, was initiated in 1998. In 2006, WHO Regional Office for Africa (WHO AFRO) Member States recommended that IHR 2005 be implemented using the IDSR framework. The IDSR strategy is aimed at integrating the collection, analysis, and reporting of data on 40 priority diseases and conditions at different levels of the health system, including relevant laboratory data. Over the past decade, the IDSR strategy has successfully reduced parallel disease-specific surveillance programmes, increased the capacity of central laboratories, and unified disease reporting matrices among implementing countries in the African region. It has enhanced adherence to IHR by encouraging and establishing early disease warning and real-time event management systems at the national level, including prompt reporting to the WHO through national IDSR focal point.

IHR core capacity target dates have been revised twice, first to 2014 and then to 2019 after the Ebola virus disease outbreak in West Africa. As of 2014, two-thirds of countries had not met their core capacity requirements and 48 countries had not responded to WHO queries with respect to their state of readiness.^13,14^ In hindsight, such shortcomings may have contributed to the state of unpreparedness within the West African sub-region that increased vulnerability to the recent Ebola virus disease outbreaks.

Recent successes in efforts to strengthen African laboratory networks, laboratory infrastructure, rapid diagnostic technologies, and quality management systems need to be comprehensively integrated into the core functions of the IDSR through new regional structures or coordination mechanisms. Furthermore, enhanced research and development support through already-existing public health research institutes and centres of excellence would not only provide inter-disciplinary technical skills and capacity to staff for quality services and accurate diagnosis, but also assist in the systematic introduction of new and rapid diagnostic technologies and the provision of periodic epidemiological research surveys that will inform efficient planning for health services. Surveillance mechanisms need high-level coordination at the sub-regional and national levels, with frequent modification.^15^ Provision of this level of coordination for active surveillance, timely analyses and response, especially for events of public health significance, are anticipated through the establishment of the Centres for Disease Control and Prevention in Africa.^16^

## Global Health Security Agenda

The Global Health Security Agenda (GHSA), formed in 2014 in response to the Ebola virus disease crisis and supported by high-level international collaboration, is a multi-country concerted effort toward creating a world safe and secure from infectious disease threats.^17^ To effect coordination, 11 action packages were designed and entrusted to contributing countries for enlisting political leadership and action. Action packages are bound by a Prevent–Detect–Respond framework. There is a need to develop in-country mechanisms and responsibilities to address the GHSA. Integration of health systems across diseases and structures would provide a path for this. It is envisaged that the capacity to respond to the objectives of the GHSA would depend on maximising detection and surveillance capabilities through integrated laboratory frameworks within target countries.

## Integration of laboratory networks, surveillance and public health institutions

**FIGURE 1:**
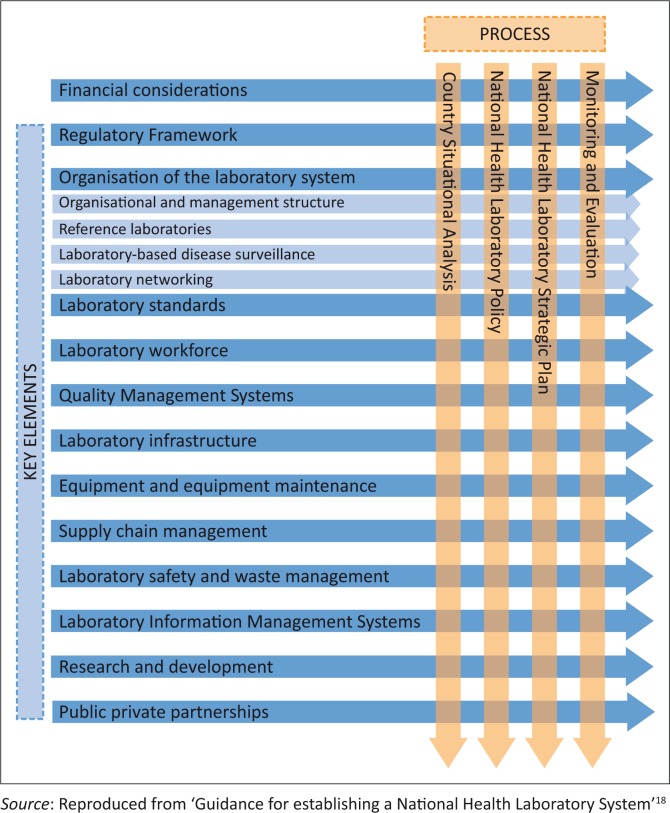
Key crosscutting elements of a National Health Laboratory System.

Integration envisages a process whereby regional and country-level governmental services, including multi-dimensional functions such as health financing, human resources, strategic planning, and others, along with international developmental partners and donors, work closely to promptly reach and sustain the goal of health security for all. In the resource-limited settings of Africa, integrating and coordinating laboratory services and different service components of national health structures would lead to efficient use of resources and building of laboratory networks, as well as ensuring that laboratories contribute to national disease surveillance and control at all levels of health services (Figure 1).^18^

**FIGURE 2:**
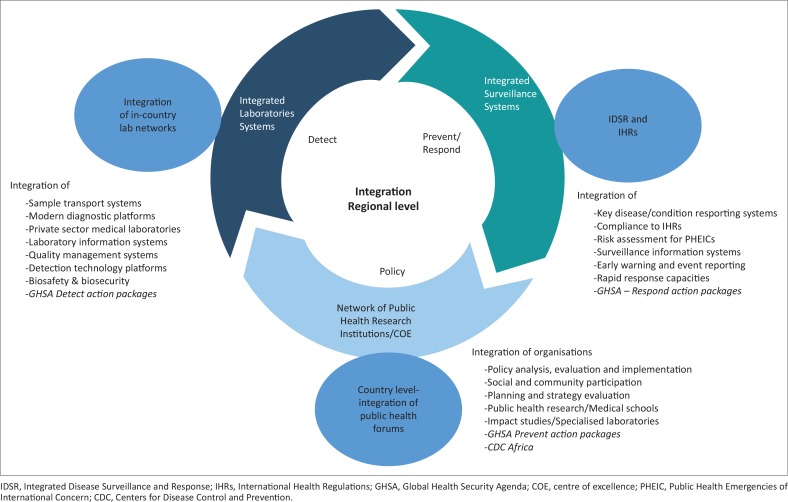
Integration as conceptual schema with three key components – laboratory networks, surveillance systems, and public health institutions. Several elements of each component are listed for integration to achieve the goal of public health laboratories. This helps with prompt adjustment to policy changes and timely detection through accurate technologies, and ensures prevent/respond measures against diseases. Achieving national and regional collaboration enhances countries’ capacities to meet the Integrated Disease Surveillance and Response and International Health Regulations targets.

In principle, integration of laboratory services should be achieved first at the country level, followed by the sub-regional level, as integration aims to best utilise the available resources in an unbiased fashion. Figure 2 depicts a conceptual, regional integration schema with functions. To achieve the desired goal, complete structural, operational and functional cohesion are required. In practice, this could be achieved by forging multi-level coordination, including appointment of officers with sufficient authority to oversee and coordinate functions in line with a defined strategy at the country and sub-regional levels. At the sub-regional level, the coordination mechanism will encompass intergovernmental bodies, relevant functions within sub-regional economic blocs, and partners. Country- and regional-level functionaries would jointly develop a response strategy to ensure that planned activities are implemented. A concerted effort to integrate modern diagnostic platforms, specifically those that use a single piece of equipment for diagnosis of multiple diseases, are needed within this regional schema. Information gathering, analyses, monitoring and evaluation of activities will be part of the action package and response at this level. The WHO and partners, including the US Centers for Disease Control and Prevention, the U.S. President’s Emergency Plan for AIDS Relief, the African Society for Laboratory Medicine, the African Union and others, would provide oversight for country–country cooperation and ensure that networks of laboratories, surveillance systems and public health institutes are functional. The schema proposes integration of the GHSA packages at the country and sub-regional levels. While it is proposed to entrust relevant disease and disorder detection capabilities to the integrated laboratory networks, response would be entrusted to the IDSRs, and prevention to public health institutions.

## Conclusion

The Ebola virus disease crisis has reinforced the need to act decisively on a new regional and global structure, as well as the necessity for integrating in-country and regional health systems under one unified, rapid service delivery mechanism, with early warning, detection and response capabilities. Political will and fiscal and administrative commitments are urgently required from governments to ensure that cross-cutting integration of health systems happens and that the IHR core requirements and the GHSA are met.
